# Interpersonal Psychotherapy to Reduce Psychological Distress in Perinatal Women: A Systematic Review

**DOI:** 10.3390/ijerph17228421

**Published:** 2020-11-13

**Authors:** Katherine S. Bright, Elyse M. Charrois, Muhammad Kashif Mughal, Abdul Wajid, Deborah McNeil, Scott Stuart, K. Alix Hayden, Dawn Kingston

**Affiliations:** 1 Faculty of Nursing, University of Calgary, Calgary, AB T2N 1N4, Canada; ksbright@ucalgary.ca (K.S.B.); elyse.lecharrois@ucalgary.ca (E.M.C.); muhammad.mughal@ucalgary.ca (M.K.M.); debbie.mcneil@albertahealthservices.ca (D.M.); 2 Alberta Health Services, Calgary, AB T2W 1S7, Canada; 3 Epidemiologist, Calgary, AB T3A 0P6, Canada; awajid@ucalgary.ca; 4 Alberta Children’s Hospital Research Institute (ACHRI), Calgary, AB T2N 4V1, Canada; 5 IPT Institute, Coralville, IA 52241, USA; iptinstitute@outlook.com; 6 Libraries and Cultural Resources, University of Calgary, Calgary, AB T2N 1N4, Canada; ahayden@ucalgary.ca

**Keywords:** systematic review, interpersonal psychotherapy, antenatal, perinatal, postpartum, women, distress

## Abstract

Background: Interpersonal psychotherapy (IPT) is a psychological intervention with established efficacy in the prevention and treatment of depressive disorders. Previous systematic reviews have not evaluated the effectiveness of IPT on symptoms of stress, anxiety, depression, quality of life, relationship satisfaction/quality, social supports, and an improved psychological sense of wellbeing. There is limited information regarding moderating and mediating factors that impact the effectiveness of IPT such as the timing of the intervention or the mode of delivery of IPT intervention. The overall objective of this systematic review was to evaluate the effectiveness of IPT interventions to treat perinatal (from pregnancy up to 12 months postpartum) psychological distress. Methods: MEDLINE(R) and Epub Ahead of Print, In-Process & Other Non-Indexed Citations and Daily (Ovid), EMBASE (Ovid), PsycINFO (Ovid), Cochrane Central Register of Controlled Trials (OVID), CINAHL with Full Text (Ebsco), Social Work Abstracts (Ebsco), SocINDEX with Full Text (Ebsco), Academic Search Complete (Ebsco), Family & Society Studies Worldwide (Ebsco), Family Studies Abstracts (Ebsco), and Scopus databases were searched from inception until 31 January 2019. Two researchers independently screened articles for eligibility. Of the 685 screened articles, 43 met the inclusion criteria. The search was re-run on 11 May 2020. An additional 204 articles were screened and two met the inclusion criteria, resulting in a total of 45 studies included in this review. There were 25 Randomized Controlled Trials, 10 Quasi-experimental studies, eight Open Trials, and two Single Case Studies. All included studies were critically appraised for quality. Results: In most studies (n = 24, 53%), the IPT intervention was delivered individually; in 17 (38%) studies IPT was delivered in a group setting and two (4%) studies delivered the intervention as a combination of group and individual IPT. Most interventions were initiated during pregnancy (n = 27, 60%), with the remaining 18 (40%) studies initiating interventions during the postpartum period. Limitations: This review included only English-language articles and peer-reviewed literature. It excluded government reports, dissertations, conference papers, and reviews. This limited the access to grassroots or community-based recruitment and retention strategies that may have been used to target smaller or marginalized groups of perinatal women. Conclusions: IPT is an effective intervention for the prevention and treatment of psychological distress in women during their pregnancy and postpartum period. As a treatment intervention, IPT is effective in significantly reducing symptoms of depression and anxiety as well as improving social support, relationship quality/satisfaction, and adjustment. Systematic Review Registration: PROSPERO CRD42019114292.

## 1. Background

The perinatal period is a time of increased social, emotional, biological, and psychological adjustments for women [1,2]. Pregnancy and the first 12 months postpartum is a developmental life stage for women which requires adjustments to changes in their physical appearance and expectations for new responsibilities [3,4]. As such, perinatal women are at increased susceptibility to psychological stress and alterations in perceived wellbeing [5,6]. Psychological distress, including stress, anxiety, and depression, resulting from pregnancy and the postpartum period is common, occurring in 15% to 25% of perinatal women [7,8]. The impact of perinatal stress, anxiety, and depression is far reaching and associated with impaired mother-fetal/infant relationships, obstetrical complications, and child cognitive-developmental problems [9,10]. Left untreated, approximately 40% of these women will have symptoms that persist until their children enter the school system [11,12]. Unfortunately, perinatal stress, anxiety, and depression often go undetected and untreated [13,14,15]. Effective treatment of perinatal mental health concerns is imperative.

Interpersonal psychotherapy (IPT) is considered a highly effective treatment for anxiety and depression [16,17,18]. Studies examining the efficacy of IPT during the perinatal period appear promising [19,20,21]. Stuart and O’Hara (1995) [22] reported that IPT is well-suited to the needs of perinatal women as IPT focuses on four areas that are significant factors in the prediction and maintenance of perinatal mental health concerns. These factors include role transitions, interpersonal disputes, grief and loss, and interpersonal deficits [22]. First, consistent with the focus of IPT, role transitions associated with becoming parents correlate with perinatal mental health symptoms and resulting interpersonal relationship disputes [23,24,25]. Secondly, interpersonal disputes are one of the most significant stressors for couples during the perinatal period. Next, grief and loss during the perinatal period are also a focus of IPT [22]. Finally, interpersonal deficits, in particular low social support and marital discord, are strongly associated with perinatal anxiety and depression [26,27].

IPT is an intervention aimed at alleviating psychological symptoms, coping with problems due to loss, change, and relationship conflict, thereby improving interpersonal functioning [25,28]. It is based on the concept that when faced with adversity, factors such as attachment styles, communication patterns, and the quality of social support networks contribute considerably to an individual’s range of symptoms of psychological distress [25]. Conceptualizations of social supports come from work on attachment theory, trust, and coping in times of adversity [29]. These social supports play an important role in how individuals navigate the coping process and manage stress [30,31]. Social supports vary in type and can include emotional support, practical help, social companionship, and motivational support [32]. Emotional support offers reassurance about individuals’ self-worth, unconditional positive regard, and the opportunity for confiding [29,32]. Practical help, also known as instrumental or tangible support, provides direct assistance [32,33]. Social companionship is important as it facilitates individuals engaging in leisure activities [32]. Motivational support is defined as help that supports an individual’s plan or goals [34]. IPT endeavours to improve attachment security, interpersonal change, and psychological distress [25,35] as a mechanism for improving individual coping and resilience.

In addition to the four salient areas of focus, IPT is consistent with many women’s desire to self-manage their mental health concerns [36,37]. While the literature suggests that psychological therapy is effective, perinatal women report significant barriers to seeking psychological support. These barriers include stigma (self and by their healthcare professional), uncertainty about whether their symptoms are normal or abnormal, inability to articulate their distress, wanting the opportunity to self-manage first, not wanting to take psychotropic medications, lack of time, financial expenditure, location and proximity of services, transportation issues, and challenges associated with childcare [8,38,39]. Therefore, instead of using formal treatments, women are more inclined to seek the informal support of friends and family, printed material, or computer/web-based intervention programs [7,8,40].

In a recent systematic review (2018) looking at the efficacy of IPT in perinatal women, 28 studies endorsed the effectiveness of IPT in the prevention and/or treatment of perinatal distress [41]. However, the review lacked adherence to systematic literature review best practices as the search was limited to two databases, screening was completed by only one reviewer, and the search strategy included limited keywords, did not include variations of terms as hyphenated terms (e.g., peri-natal), and did not include subject headings [41]. As such, clinicians, researchers, and decisionmakers would benefit from a systematic, comprehensive, and transparent approach to examining the use of IPT in perinatal women.

The goal of the current systematic review was to synthesize the current literature, evaluating the effectiveness, feasibility, and acceptability of IPT interventions to treat perinatal psychological distress. The question guiding this systematic review was: What is the effectiveness of IPT for women during the perinatal period on the reduction of stress, anxiety, and depression and improvement in quality of life, relationship satisfaction/quality, social support, and psychological wellbeing?

## 2. Methods

### 2.1. Protocol and Registration

The protocol for this systematic review was developed based on the Preferred Reporting Items for Systematic reviews and Meta-analyses Protocols (PRISMA-P) [42] and has been registered with PROSPERO CRD42019114292. The systematic literature protocol paper has also been published (K. S. Bright et al., 2019) [43].

### 2.2. Eligibility Criteria

The studies selected for inclusion in this systematic review met the following eligibility criteria, which are described according to participants, study design (including publication, language, and year), intervention, and outcomes.

### 2.3. Participants

Perinatal women from conception to 12 months postpartum who participated in an IPT intervention were included. For this systematic review, we excluded women who were not pregnant or postpartum.

### 2.4. Study Design

The review considered studies evaluating the feasibility, acceptability, effectiveness, and/or efficacy of IPT in perinatal women. Experimental studies such as randomized controlled/clinical trials (RCTs), quasi-experimental studies, as well as single group pre-post studies were included in the review. Observational studies, including cohort and case control studies, were included. We included qualitative studies that explored the acceptability of IPT interventions. We excluded conference papers, dissertations, reviews, and non-English publications.

### 2.5. Interventions

We defined IPT intervention as interpersonal therapy, or any intervention, counseling, psychotherapy, therapy, or program where there was a component of IPT offered. IPT included those interventions targeted towards women during the perinatal period.

### 2.6. Comparator

We included studies with all types of comparator groups, such as pre-post interventions, non-exposed control group, or a group exposed to a different intervention.

### 2.7. Information Sources and Search Strategy

MEDLINE(R) and Epub Ahead of Print, In-Process & Other Non-Indexed Citations and Daily (Ovid), EMBASE (Ovid), PsycINFO (Ovid), Cochrane Central Register of Controlled Trials (OVID), CINAHL with Full Text (Ebsco), Social Work Abstracts (Ebsco), SocINDEX with Full Text (Ebsco), Academic Search Complete (Ebsco), Family & Society Studies Worldwide (Ebsco), Family Studies Abstracts (Ebsco), and Scopus databases were searched from database inception to 31 January 2019 and rerun on 11 May 2020. (See Supplementary Materials for the Medline search strategy).

### 2.8. Screening of Studies

**Figure 1:**
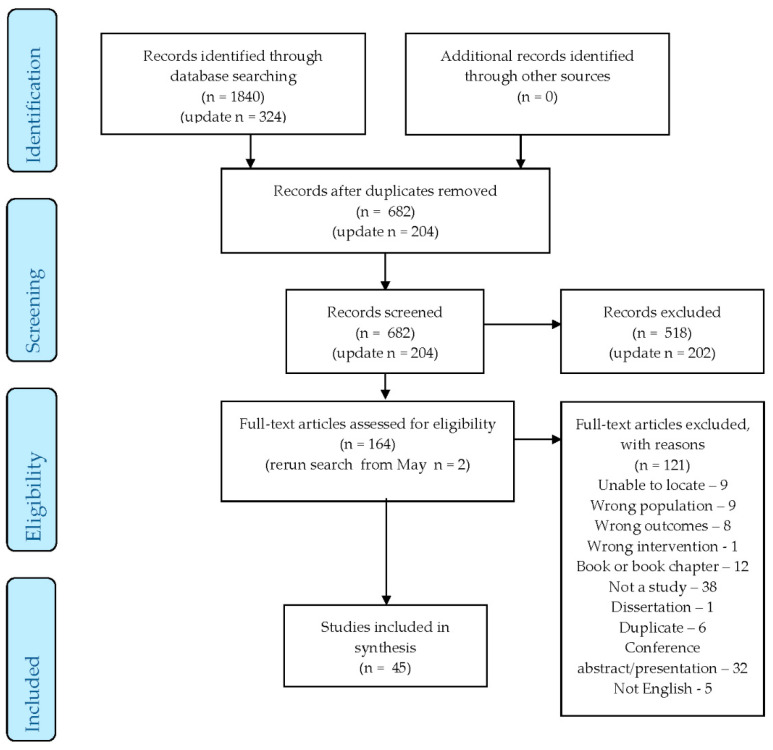
PRISMA (Preferred Reporting Items for Systematic Reviews and Meta-Analyses) Diagram.

Prior to screening, the two reviewers (KSB and EMC) completed a calibration exercise where 10% of studies were reviewed independently and then together assessed for inter-rater agreement. In the calibration exercise, there was 93% agreement. Following the calibration exercise, the two reviewers independently screened the studies for eligibility in two steps. The first step consisted of reviewing all studies’ titles/abstracts to identify studies that met the eligibility criteria. The second step consisted of reviewing the provisionally included studies’ full text to ensure that they met all the inclusion criteria. Any disagreements were resolved by discussion between the two reviewers. There were 45 studies that met the inclusion criteria (Figure 1. PRISMA Diagram).

### 2.9. Risk of Bias in Individual Studies

Studies were included regardless of methodological quality. The Effective Public Health Practice Project (EPHPP) Quality Assessment Tool was used for quality assessment. Two reviewers (KSB and EMC) independently assessed all studies for quality and disagreements were resolved by discussion between the two reviewers.

## 3. Results

### 3.1. Characteristics of Included Studies

Characteristics of the 45 studies included in this systematic review are presented in Table 1. There were 25 (56%) RCTs, 10 (22%) quasi-experimental studies, eight (18%) open trials, and two (4%) single cases. Of these studies, 33 (73%) provided IPT as a treatment and 12 (27%) provided it for prevention. Most studies (n = 28, 62.2%) were conducted in the USA, with 11.1% (five) in China, 6.7% (three) in Australia, 4.4% (two) in Canada, 4.4% (two) in Hong Kong, 2.2% (one) in Iran, 2.2% (one) in Singapore, 2.2% (one) in Austria, 2.2% (one) in Hungary, and 2.2% (one) in Israel. Among the RCTs and quasi-experimental studies, 15 (48.6%) used comparisons of treatment as usual (TAU), 16 (37.1%) were active treatment, three (8.6%) were waitlist control (WLC), one (2.9%) was WLC and TAU, and one (2.9%) was WLC and active treatment. Of the active treatment comparison type studies, six studies used education-based programs, four studies used psychological programs/sessions, two studies used antidepressant medications, and one used mindfulness-based therapy.

Characteristics of the interventions are presented in Table 2. In most studies (n = 24, 53%), the IPT intervention was delivered individually; in 17 (38%) studies IPT was delivered in a group setting, two (4%) studies delivered the intervention as a combination of group and individual IPT, and two (4%) studies included partners in the delivery of the intervention. Most studies (n = 29, 64.4%) delivered the IPT face-to-face, while two (4.4%) studies delivered IPT over the phone and 14 (31.1%) studies combined face-to-face and telephone calls.

Most interventions were initiated during pregnancy (n = 27, 60%), with the remaining 18 (40%) studies initiated during the postpartum period. IPT was administered individually in 24 (53%) studies and in groups in 17 (38%) studies. Women’s partners were included in the intervention in two (4%) studies. Most studies (n = 30, 66.7%) provided IPT in a community setting (e.g., women’s recreation facility), 12 (26.7%) studies provided IPT in the clinical setting (e.g., prenatal clinic), and three (6.7%) studies provided IPT in a mixed clinical and community setting. The number of IPT sessions ranged from two to 16 sessions, with an average of eight sessions. Most studies (n = 35, 78%) reported provided IPT according to a study or intervention protocol.

Characteristics of the method of assessment for outcomes are presented in Table 3. In most studies (n = 28, 62.2%), depressive symptoms were assessed using the Edinburgh Postnatal Depression Scale (EPDS), while 16 (35.6%) studies used the Hamilton Depression Rating Scale (HAM-D), 16 (35.6%) used the Beck Depression Inventory (BDI), three (6.7%) studies used the CESD, and three (6.7%) studies used the SCL-20. Symptoms of anxiety were assessed in 18 (40%) studies, most commonly using the State-Trait Anxiety Inventory and Beck Anxiety Inventory. Stress levels were assessed in 10 (22%) of the studies. Maternal-infant attachment was assessed in 16 (36%) of the studies. Eleven (24%) of the studies assessed social support. Relationship satisfaction/quality was assessed in 17 (38%) of the studies.

Characteristics of study methodological quality are presented in Table 4. Methodological quality was assessed using the Effective Public Health Practice Project (EPHPP) Quality Assessment Tool [86]. The study scores ranged from 1 (strong) to 3 (weak), with an average of 2 (moderate). There were 18 studies (40%) categorized as strong overall, 14 (31%) studies were moderate overall, and 13 (29%) studies were weak overall. Study design was assessed as strong in 26 (57.8%) studies, intervention integrity was determined to be strong in 35 (78%) studies, and data analysis was assessed as strong in 20 (44%) studies.

Among the studies that reported sample demographic characteristics, maternal age ranged from 18 to 38 years old with a mean age of 30 years. The average gestational age for pregnant women ranged from six to 40 weeks, with an average of 23.7 weeks. The weeks postpartum of participants ranged from 0.5 to 96 weeks postpartum, with an average of 24.4 weeks.

### 3.2. Prevention Studies

Among the 13 prevention studies, 12 (92%) were delivered during pregnancy and one (8%) was delivered in the postpartum period.

### 3.3. Treatment Studies

Among the 33 treatment studies, 16 (48.5%) were delivered during the prenatal period and 17 (51.5%) studies were delivered in the postpartum period.

### 3.4. Change in Depressive Symptoms Between Treatment and Comparison Groups

Twelve prevention studies aimed to reduce the risk of depression in participants receiving IPT. Five studies [45,55,56,59,67] reported a significant reduction of depressive symptoms levels over time. These improvements were small to moderate in magnitude. No studies had large effect sizes. Reductions in depressive symptoms were also significantly larger in studies where IPT was delivered in a group format compared to individual IPT.

Thirty-two (71%) treatment studies assessed change in depressive symptoms among participants receiving IPT. Twenty-six studies reported a significant improvement in depressive symptoms over time. The improvements were determined to be in the moderate to large range. Reductions in depressive symptoms were more common in studies where the interventions were initiated in the postpartum period than in studies where interventions were initiated during pregnancy.

### 3.5. Change in Anxiety Symptoms Between Treatment and Comparison Groups

Seven prevention studies aiming to reduce the risk of symptom levels of anxiety addressed the change in symptoms of anxiety among participants receiving IPT. One study (Bowen et al., 2014) reported a significant reduction in the risk level of anxiety symptoms. The effect size of the intervention on symptoms of anxiety was not reported in this study.

Eleven treatment studies assessed the change in symptoms of anxiety among participants receiving IPT. Six studies [47,52,53,60,62,69] reported significant reductions in symptoms of anxiety. There was an overall reduction in symptoms of anxiety among participants receiving IPT, with an effect size in the moderate range. More studies of individual delivery showed a reduction in anxiety than group delivery. Reductions in anxiety were also noted more frequently in studies where IPT was delivered in a medical/clinical setting compared to a community setting.

### 3.6. Change in Stress Symptoms Between Treatment and Comparison Groups

Three prevention studies aimed at reducing the risk of stress levels assessed change in symptoms of stress among participants receiving IPT. Two studies (Bowen et al., 2014; Leung & Lam, 2012) reported a significant reduction in the risk of symptom levels of stress. One study did not report an effect size of the intervention and the other reported a very small effect size (Leung & Lam, 2012).

Seven treatment studies assessed for change in symptoms of stress among women receiving IPT. Two of these studies (Field et al., 2009; Field et al., 2013) reported a significant reduction in symptoms of stress for participants receiving IPT. The effect sizes of the intervention were not reported.

### 3.7. Change in Relationship Quality Between Treatment and Comparison Groups

Three prevention studies aiming to reduce the risk of relationship distress assessed relationship quality/satisfaction among participants receiving IPT. There were no studies that reported a significant improvement in relationship quality/satisfaction.

Twelve treatment studies assessed relationship quality/satisfaction among women receiving IPT. Four studies (Chung, 2015; Field et al., 2013; Hajiheidari et al., 2013; Mulcahy et al., 2010) reported significant improvements in relationship quality, with an effect size in the small range. Studies with married/cohabitating participants were more likely to have greater improvements in their relationship quality than those women without partners.

### 3.8. Change in Social Support Between Treatment and Comparison Groups

Four prevention studies aiming to reduce the risk of distress related to poor support assessed social support among participants receiving IPT. Three of these studies (L. L. Gao et al., 2012; L. L. Gao et al., 2012; L. L. Gao et al., 2015) reported significant improvements in social support. The effect size was in the small range.

Seven treatment studies assessed the change in social support among participants receiving IPT. Three studies (Lenze & Potts, 2017; Lenze et al., 2015; Mulcahy et al., 2010) reported significant improvements in social support. The effect size was in the medium to large range. Studies with participants who had higher levels of education were more likely to experience significant improvements in social support.

### 3.9. Change in Attachment Levels Between Treatment and Comparison Groups

There were no prevention studies that assessed attachment. There were eight treatment studies that assessed attachment among participants receiving IPT. Three of these studies (Mulcahy et al., 2010; Posmontier et al., 2019; Spinelli, Endicott, Leon, et al., 2013) reported significant improvements in attachment. While these improvements were reported to be statistically significant, the effect size of the IPT intervention was not reported.

### 3.10. Change in the Level of Adjustment Between Treatment and Comparison Groups

There was one prevention study aiming to reduce the risk of poor adjustment that assessed for adjustment among participants receiving IPT. This one study (Crockett et al., 2008) reported that the level of adjustment was statistically significant only between 2–3 weeks and 3 months postpartum. No effect size was reported.

There were 12 treatment studies that assessed for level of adjustment among participants receiving IPT. There were no studies that reported any significant improvements in level of adjustment.

## 4. Discussion

This review of the literature provides evidence that IPT is an effective intervention for the prevention and treatment of psychological distress in women during their pregnancy and postpartum period. As a preventive intervention, IPT is superior to comparison conditions, including active interventions, treatment-as-usual, and no intervention, for reducing the risk of depression. As a treatment intervention, IPT is effective in significantly reducing symptoms of depression and anxiety as well as improving social support, relationship quality/satisfaction, and adjustment. IPT is superior to comparison conditions including active interventions, treatment-as-usual, and no intervention for reducing depressive symptoms as well as improving social support and relationship quality.

There is evidence supporting the use of IPT to prevent depression in perinatal women. These findings suggest that IPT is effective as both a prevention intervention and for those women at high risk due to the presence of risk factors including a previous diagnosis of depression (Zlotnick et al., 2006) or post-traumatic stress disorder (PTSD) (Grote et al., 2015; Grote et al., 2017; Zlotnick et al., 2011). There was one preventive study that reported outcomes for symptoms of anxiety (Bowen et al., 2014). This study found that IPT was effective in reducing anxiety symptoms and worry over time in pregnant women compared to active interventions, treatment-as-usual, and no interventions. Given the far reaching impact of prenatal anxiety on women and their children (Brunton, et al, 2015 [87]; Mughal et al., 2019 [88]; Brunton, Dryer, Field, 2017 [89]; K. Bright & Becker, 2019 [90]), future research exploring preventive interventions in prenatal women would benefit from including assessment of anxiety in addition to depressive symptoms. There is a need for investigating the diagnostic outcomes of anxiety and anxiety-related disorders, including the prevalence of perfectionism and obsessive-compulsive disorder, as preliminary work in this area suggests that there is increased risk for these disorders during the perinatal period (Kane, Winton, Eliot, & McEvoy, 2017 [91]; Lowndes, Egan, & McEvoy, 2019 [92]; Standeven, Nestadt, & Samuels, 2020 [93], Buchholz, Hellberg, & Egan, Abramowitz, 2020 [94];).

In this review, group prevention interventions resulted in greater reduction in risk of symptom levels of depressive than individually administered interventions. Groups have a valuable set of therapeutic characteristics where women are provided with a supportive network of peers with shared feelings, thoughts, and problems (Marmarosh, Holtz, & Schottenbauer, 2005) [95]. Women gain insight into the universality of their problems, which helps to normalize their experiences (Reay et al., 2006). Group therapy allows women to increase their coping strategies, knowledge, and skill through vicarious learning. Helping others solve their problems can increase their sense of competence. It may also be that the social skills and competencies gained through group-based IPT prevent the onset of depressive symptoms by specifically moderating relationship challenges.

While RCTs of IPT for mental health disorders show a moderate to large effect on depression compared with control groups, IPT has not been found to be more effective than other psychotherapies such as CBT for depression (Cuijpers, Donker, Weissman, Ravitz, & Cristea, 2016 [96]; Jakobsen, Hansen, Simonsen, Simonsen, & Gluud, 2012 [96]). Research does suggest that pharmacotherapy may be mildly more effective than psychotherapies (Cuijpers et al., 2016 [97]; Cuijpers, van Straten, Andersson, & van Oppen, 2008 [98]). When pharmacotherapy is combined with psychotherapy, it is not more effective than pharmacotherapy alone, but is more effective than IPT alone (Cuijpers et al., 2016 [96]; Nillni, Mehralizade, Mayer, & Milanovic, 2018 [99]).

There was a trend that more studies of individually administered IPT showed a reduction of anxiety symptoms than group offered IPT. Individual therapy has the advantage of participants receiving greater attention to their individual issues, closer monitoring of symptoms, and more tailored adaptation of the intervention to issues that are particularly relevant to the individual (O’Shea, Spence, & Donovan, 2015) [100]. Previous literature reviews and meta-analyses have obtained contradictory findings (Cuijpers et al., 2008; Goodman & Santangelo, 2011; L.E. Sockol, Epperson, & Barber, 2011; L. E. Sockol, Epperson, & Barber, 2013) [97,101,102,103]. Future preventive and treatment research would benefit from including assessment of acceptability of group and individual therapy. Investigation of potential predictors of treatment efficacy should include a history of depressive disorders and anxiety-related disorders as well as their comorbidity to determine if these characteristics are associated with delivery method and differential efficacy.

In six RCTs examining the effect of IPT on anxiety, compared to other psychotherapies, this resulted in a small nonsignificant difference in favour of the alternative therapies such as CBT over IPT (Cuijpers et al., 2016; Nillni et al., 2018) [96,99]. There is one study investigating the effect of paroxetine and CBT compared to CBT alone and it was found that there was no significant difference between groups (Misri, Reebye, Corral, & Mills, 2004) [104]. Given the paucity of research in this area, this is concerning given that anxiety symptoms and comorbid symptoms are prevalent in perinatal women, therefore it is important that there is further research on effective treatments.

### 4.1. Strengths

There are numerous strengths of this systematic review, which include explicit methods description and comprehensive database searches to methodologically search for articles exploring the use of IPT/IPT-based interventions in the perinatal population. This transparent and systematic approach to reviewing the literature included the use of a librarian for the search and two reviewers with content expertise for the assessment of inclusion and data extraction attempted to reduce reviewer bias. This rigorous process facilitates a reproducible and objective criteria to select relevant studies and adequately assess their quality.

### 4.2. Limitations

A major limitation of the studies evaluating IPT, whether for prevention or treatment, is that few studies addressed outcomes such as social support, relationships, and adjustment the same way. Improving these interpersonal areas are among the goals of IPT. As such, there needs to be consistency in how these elements are operationalized in a perinatal population. Implications for future IPT intervention studies involve assessing perinatal women’s change in interpersonal functioning and involving women’s partners in treatment.

Findings from this review of IPT in perinatal women are limited to IPT being delivered face-to-face or via telephone. Literature examining online IPT in non-perinatal populations suggests that despite high dropout rates, internet-delivered self-guided IPT is effective in reducing depressive symptoms (Donker et al., 2013). Future research requires well-designed RCTs that compare internet-delivered IPT to active, treatment-as-usual, and no treatment. Additionally, internet-based IPT trials will need to assess differences in prevention versus treatment, prenatal versus postpartum women, and group versus individual treatment.

This review is limited by the lack of detailed descriptions of recruitment and retention strategies of the individual studies. Further limitations include the inclusion/exclusion criteria of reviewing only English-language articles, which may reduce generalizability to non-English speaking populations. Similarly, this review included only peer-reviewed literature and excluded government reports, dissertations, conference papers, and reviews. This limited the access to grassroots or community-based recruitment and retention strategies that may have been used to target smaller or marginalized groups of perinatal women.

### 4.3. Research Implications

Further studies would benefit from refinement of the perinatal IPT treatment. In future studies, the IPT intervention will need to include a comprehensive IPT manual to promote adherence/competence measures. Perinatal IPT research will also benefit from development of far-reaching training programs for those delivering IPT in research, community, and clinical settings. Improving the structure of IPT and training of clinicians who can deliver evidence-based IPT has the potential to improve outcomes for perinatal women.

Additional research is required to evaluate the efficacy of internet-based treatment compared to telephone and face-to-face delivery. Regardless of the type or mode of delivery, research aimed at exploring the mechanisms of action is necessary for IPT interventions. This will aid in further refining IPT interventions, improving outcomes, and determining whether the intervention is applicable in additional settings.

Studies exploring various techniques for keeping women engaged in treatment for extended periods of time are warranted to ensure that perinatal women can complete the full IPT intervention. This will take into consideration an individual’s preference for treatment. Longitudinal studies of different intervention models (varying in length and delivery) and social support are needed. More research into how IPT interventions can be implemented as a part of routine prenatal care is needed.

### 4.4. Clinical Implications

There is a large body of research that demonstrates the effectiveness of treatments for depression and anxiety during the perinatal period (Milgrom, Negri, Gemmill, McNeil, & Martin, 2005; Nillni et al., 2018; L. E. Sockol, 2018; L. E. Sockol et al., 2013) [99,101,102,105]. Given that there is strong evidence for and no difference in the effectiveness for prevention and treatment of various psychotherapies allows for women to determine which psychotherapy they would choose. This choice may also be influenced by the mental health services offered through the trained therapists in their area. Additionally, the decision on whether to use pharmacotherapy in addition to psychotherapy during the perinatal period is complex and requires the consideration of many factors, including the effects of untreated maternal mood and/or medication exposure on both maternal and fetal outcomes. Clinical discussion making around mental health treatment options would benefit from thoughtful conversations between clinicians and the perinatal women as well as their families as no one treatment works for everyone.

## 5. Conclusions

This systematic review provides evidence that IPT is an effective intervention for the prevention and treatment of psychological distress in women during their pregnancy and postpartum period. This review also highlights the need for robust, high quality RCTs exploring different intervention models for women during the perinatal period.

## Figures and Tables

**Table 1:** Characteristics of Included Studies.

Study	Number (N)	Country	Trial Type (OT, RCT, QRT)	Study Type (Prevention or Treatment)	Comparison Type (Active, Treatment as Usual, Waitlist Control)	Comparison Treatment	Sample Population (Community, Clinical, Mixed, Prenatal, Postpartum)	Inclusion Type (Clinical Diagnosis, Self-Reported, Selected/Indicated, Universal)	Effectiveness of Treatment on Psychological Wellbeing
Bhat et al. (2017) [44]	160	USA	RCT	Treatment	Treatment as usual	Treatment as usual (Maternity support services (MSS) Plus)	Community, Prenatal	Selected/Indicated	Yes—depressive symptoms
Bowen, Baetz, Schwartz, Balbuena, and Muhajarine (2014) [45]	106	Canada	QRT	Prevention	Active	Mindfulness-Based Therapy (MBT)	Community, Prenatal	Universal	Yes—depressive symptoms and stress
Brandon et al. (2012) [21]	11	USA	OT	Treatment			Clinical, Mixed Prenatal and Postpartum	Clinical diagnosis	Yes—depressive symptoms
Chen (2011) [46]	176	Singapore	QRT	Treatment	Active	Psychological, occupational, and/or medical social worker community resources program	Clinical, Postpartum	Self-reported	
Chung (2015) [47]	1	Hong Kong	Single Case Design	Treatment			Clinical, Postpartum	Clinical diagnosis	Yes—depression and anxiety symptoms
Clark, Tluczek, and Wenzel (2003) [48]	66	USA	QRT	Treatment	Active, Waitlist Control	Mother-Infant Therapy Group (MIT-G), Waitlist Control Group (WLC)	Clinical, Postpartum	Universal	Yes—depressive symptoms and stress
Crockett, Zlotnick, Davis, Payne, and Washington (2008) [49]	36	USA	RCT	Prevention	Treatment as usual	Standard Antenatal Care	Community, Prenatal	Selected/Indicated	
Deans, Reay, and Buist (2016) [50]	1	AUS	Single Case Design	Treatment			Community, Postpartum	Clinical diagnosis	
Dennis, Grigoriadis, Zupancic, Kiss, and Ravitz (2020) [51]	241	Canada	RCT	Treatment	Treatment as usual	Treatment as usual (Standard postpartum depression services)	Community, Postpartum	Selected/Indicated	
Field et al. (2009) [52]	112	USA	QRT	Treatment	Active	Group Interpersonal psychotherapy (IPT) and Group IPT and Massage Therapy	Community, Prenatal	Clinical diagnosis	Yes—depression, anxiety, and stress
Field, Diego, Delgado, and Medina (2013) [53]	44	USA	RCT	Treatment	Active	Peer support versus group IPT	Community Prenatal	Clinical diagnosis	Yes—depression, anxiety, and stress
Forman et al. (2007) [54]	176	USA	RCT	Treatment	Waitlist Control (depressed mothers) and Comparison Group (non-depressed mothers)	Waitlist control (WLC) and Control group (CG) (videotaped tasks to measure infant emotionality and parenting), Waitlist control (IPT for 12 weeks started after IPT group received their 12 weeks of IPT)	Community, Postpartum	Clinical diagnosis	Yes—depressive symptoms
L. L. Gao, Chan, Li, Chen, & Hao (2010) [55]	194	China	RCT	Prevention	Active	Childbirth education program only (routine antenatal education, consisting of 2 × 90-min sessions conducted by midwives, content: delivery process and childcare)	Community, Prenatal	Universal	Yes—depressive symptoms
L. L. Gao, Chan, & Sun, 2012 [56]	194	China	RCT	Prevention	Active	Childbirth education program only (routine antenatal education, consisting of 2 × 90-min sessions conducted by midwives, content: delivery process and childcare)	Community, Prenatal	Universal	Yes—depressive symptoms
L. L. Gao, Luo, and Chan (2012) [57]	83	China	OT	Prevention			Community, Postpartum	Universal	
L. L. Gao, Sun, and Chan (2014) [58]	68	China	QRT	Prevention	Active	Childbirth education program only (routine antenatal education, consisting of 2 × 90-min sessions conducted by midwives, content: delivery process and childcare)	Community, Prenatal	Universal	
L. L. Gao, Xie, Yang, and Chan (2015) [59]	180	China	RCT	Prevention	Treatment as usual	Treatment as usual (TAU) (pamphlet on sources of assistance after discharge)	Community, Postpartum	Universal	Yes—depressive symptoms
Grote, Bledsoe, Swartz, and Frank (2004) [60]	12	USA	OT	Treatment			Community, Prenatal (pregnant, depressed, socioeconomically disadvantaged)	Self-reported	Yes—depression and anxiety symptoms
Grote et al. (2009) [61]	53	USA	RCT	Treatment	Treatment as usual	Enhanced Usual Care	Community, Prenatal (pregnant, depressed, socioeconomically disadvantaged)	Self-reported	Yes—depressive symptoms
Grote et al. (2015) [62]	164	USA	RCT	Treatment	Active	Intensive Maternity Support Services (MSS-Plus)	Community, Prenatal (pregnant, depressed, socioeconomically disadvantaged)	Self-reported	Yes—depression and anxiety symptoms
Grote et al. (2017) [63]	164	USA	RCT	Treatment	Active	Intensive Maternity Support Services (MSS-Plus)	Community, Prenatal (pregnant, depressed, socioeconomically disadvantaged)	Self-reported	Yes—depressive symptoms
Hajiheidari, Sharifi, and Khorvash (2013) [64]	34	Iran	QRT	Treatment	Treatment as usual	Referred to Mental health providers	Community, Postpartum	Clinical diagnosis	Yes—depressive symptoms
Kao, Johnson, Todorova, and Zlotnick (2015) [65]	99	USA	RCT	Treatment	Treatment as usual	Treatment as usual (TAU) (Standard care—optional classes on breastfeeding, infant safety, and parenting—no depression assessments or mental health groups)	Community, Prenatal	Selected/Indicated	
Klier, Muzik, Rosenblum, and Lenz (2001) [66]	17	Austria	OT	Treatment			Clinical, Postpartum	Clinical diagnosis	Yes—depressive symptoms
Kozinszky, Dudas, Devosa, Csatordai, Tóth, et al. (2012) [67]	1719	Hungary	RCT	Prevention	Treatment as usual	Treatment as usual (TAU) (4 group meetings: education on pregnancy, childbirth, and baby care)	Community, Prenatal	Universal	Yes—depressive symptoms
Lenze, Rodgers, and Luby (2015) [68]	9	USA	OT	Treatment			Community, Prenatal	Clinical diagnosis	Yes—depressive symptoms
Lenze and Potts (2017) [69]	42	USA	RCT	Treatment	Treatment as usual	Treatment as usual (TAU) (Enhanced Treatment as Usual)	Community, Prenatal	Clinical diagnosis	Yes—depressive and anxiety symptoms
Leung and Lam (2012) [70]	156	Hong Kong	RCT	Prevention	Treatment as usual	Routine antenatal care from MCHC (physical exam and brief individual interview)	Community, Prenatal	Universal	Yes—stress
Moel, Buttner, O’Hara, Stuart, and Gorman (2010) [71]	176	USA	RCT	Treatment	Waitlist control and Treatment as usual	Treatment as usual (TAU) (no depression, no intervention), Waitlist control (no intervention during 12 week wait, then received 12-week IPT)	Community, Postpartum	Selected/Indicated	Yes—depressive symptoms
Mulcahy, Reay, Wilkinson, and Owen (2010) [72]	57	Australia	RCT	Treatment	Treatment as usual	Encompassed all options for postnatal depression that were available to women in the Australian Capital Territory (ACT) community, such as antidepressant, natural remedies, nondirective counselling, maternal and child health nurse support, community support groups, individual psychotherapy or group therapy already provided in the community (either publicly or privately)	Clinical, Postpartum	Clinical diagnosis	Yes—depressive symptoms
Nylen et al. (2010) [73]	120	USA	QRT	Treatment	Waitlist control	Waitlist control (WLC) (after 12 week waiting period, Waitlist control received 12 IPT sessions)	Community, Postpartum	Selected/Indicated	Yes—depressive symptoms
O’Hara, Stuart, Gorman, and Wenzel (2000) [74]	120	USA	QRT	Treatment	Waitlist control	Waitlist control (WLC) (after 12 week waiting period, Waitlist control received 12 IPT sessions)	Clinical, Postpartum	Clinical diagnosis	Yes—depressive symptoms
O’Hara et al. (2019) [75]	53	USA	RCT	Treatment	Active	IPT (n = 56), Sertraline (n = 56), clinical management and pill placebo (n = 53)	Clinical, Postpartum	Clinical diagnosis	
Pearlstein et al. (2006) [76]	23	USA	QRT	Treatment	Active	Sertraline (n = 2), Sertraline and IPT (n = 10)—Sertraline component: 8 sessions over 12 weeks	Clinical, Postpartum	Clinical diagnosis	Yes—depressive symptoms
Posmontier, Neugebauer, Stuart, Chittams, and Shaughnessy (2016) [77]	61	USA	QRT	Treatment	Active	Referral to a variety of Mental Health Practitioner (MHP) who provided various psychotherapeutic modalities such as supportive and psychodynamic psychotherapy	Clinical, Postpartum	Clinical diagnosis	Yes—depressive symptoms
Posmontier et al. (2019) [78]	27	Israel	OT	Treatment	Active	Includes a variety of cognitive-behavioral, psychodynamic, psychoeducational, and/or non-specific supportive modalities, varying number, and duration of sessions	Clinical, Postpartum	Clinical diagnosis	Yes—depressive symptoms
Reay et al. (2006) [79]	18	Australia	OT	Treatment			Community, Postpartum	Selected/Indicated	Yes—depressive symptoms
M. G. Spinelli (1997) [19]	13	USA	OT	Treatment			Clinical, Prenatal	Clinical diagnosis	Yes—depressive symptoms
Spinelli and Endicott (2003) [20]	50	USA	RCT	Treatment	Active	Parenting Education Program for Unipolar Depressed Nonpsychotic pregnant women (therapist-led weekly 45 min sessions for 16 weeks)	Mixed Clinical and Community, Prenatal	Clinical diagnosis	Yes—depressive symptoms
Spinelli, Endicott, Leon, et al. (2013) [80]	142	USA	RCT	Treatment	Active	Parent Education Program (therapist-led 45 min weekly didactic lectures on pregnancy, postpartum, breastfeeding education—provided to 100% participants, and early infant development)	Mixed Clinical and Community, Prenatal	Clinical diagnosis	Yes—depressive symptoms
Spinelli, Endicott, and Goetz (2013) [81]	142	USA	RCT	Treatment	Active	Parent Education Program (therapist-led 45 min weekly didactic lectures for 12 weeks)	Mixed Clinical and Community, Prenatal	Clinical diagnosis	
Zlotnick, Johnson, Miller, Pearlstein, and Howard (2001) [82]	37	USA	RCT	Prevention	Treatment as Usual	Treatment as usual—standard medical attention and treatment provided to all attending prenatal clinic	Community, Prenatal	Selected/Indicated	
Zlotnick, Miller, Pearlstein, Howard, and Sweeney (2006) [83]	99	USA	RCT	Prevention	Treatment as Usual	Standard Antenatal Care	Community, Prenatal	Selected/Indicated	
Zlotnick, Capezza, and Parker (2011) [84]	54	USA	RCT	Treatment	Treatment as Usual	Treatment as usual—(standard medical attention and treatment provided to all attending prenatal clinic and educational material/listing of resources for IPV)	Community, Prenatal	Selected/Indicated	
Zlotnick, Tzilos, Miller, Seifer, and Stout (2016) [85]	205	USA	RCT	Prevention	Treatment as Usual	Standard Antenatal Care	Community, Prenatal	Selected/Indicated	

**Table 2:** Characteristics of Interventions.

Study	Timing (Prenatal or Postpartum)	Timing in Weeks Pregnant or Postpartum	Intervention	Comments	Methods of Administration (Individual, Partners, Groups)	Mode of Administration	Setting (Clinical or Community)	Included Partner	# of Sessions
Bhat et al. (2017) [44]	PN	MSS-Plus from pregnancy to 2 months PP; MOMCare from pregnancy to 12 months PP	Pretherapy engagement brief IPT, Pharmacotherapy or both (MOMCare)		Individual	Combination Face-to-face Telephone	Community	No	Not specified
Bowen et al. (2014) [45]	PN	15–25 weeks pregnant	IPT	6 weeks duration	Group	Face-to-face	Community	No	5 group sessions (3 groups were Mindfulness Based (MFB), 2 groups were IPT)
Brandon et al. (2012) [21]	PN	From 12 weeks prenatal to 12 weeks postpartum	1st phase—Partner assisted IPT (both partners involved, assessed depressive experience, identify and understand the triggers of depressive symptoms), 2nd phase—Role expectations (self/and partner) and quality of their interactions, 3rd phase—consolidate change, explore sources of support, and process the experience of therapy	Emotional Focused Couples Therapy (EFCT) informed—Partner-Assisted IPT	Partners	Face-to-face	Clinical	Yes	8 session to be completed within a 12-week period
Chen (2011) [46]	PP	2 weeks to 6 months postpartum	Principles of IPT and CBT		Individual, offered group support	Combination Face-to-face, telephone (high scorers who refused psychiatric intervention)	Clinical	No	Unsure of number of sessions, duration of treatment between 3–6 months
Chung (2015) [47]	PP	Unsure	IPT	Maintenance sessions—every 2 weeks for 20 min	Individual	Face-to-face	Clinical	No	12
Clark et al. (2003) [48]	PP	4–96 weeks postpartum	IPT	Three groups—IPT (Individual), M-ITG (Group, includes elements of IPT/CBT), and WLC	Individual and Group	Face-to-face	Clinical	No	M-ITG and IPT sessions: 12 (weekly for 1 h) in addition to a 1.5-h initial intake; WLC: waiting to receive M-ITG
Crockett et al. (2008) [49]	PN	24–31 weeks pregnant	ROSE Program (Reach Out, Stand Strong: Essentials for New Moms)—IPT based		Group (and Individual booster)	Face-to-face	Community (group sessions), Participant’s home (booster session)	No	4 (1.5 h during pregnancy) group sessions weekly and 1 (50 min) individual booster 2 weeks after delivery
Deans et al. (2016) [50]	PP	7 months postpartum	IPT for the mother-child relationship	Was a group intervention—reporting on one individual in the group	Group and Individual	Face-to-face	Community	Yes—1 session with partner at the halfway point (between session 5 and 6)	10 (in addition: two pre-group individual sessions and one psychoeducation partner session at the halfway point)
Dennis et al. (2020) [51]	PP	Between 2 and 24 weeks postpartum	IPT		Individual	Telephone	Community	No	12 weekly 60-min telephone IPT sessions
Field et al. (2009) [52]	PN	22–28 weeks pregnant	IPT and IPT with Massage		Group	Face-to-face	Community	No	Group IPT—1 hr per week for 6 weeks, IPT and Massage—1 hr IPT per week for 6 weeks, 20-min massage once a week for 6 weeks
Field et al. (2013) [53]	PN	22–34 weeks pregnant	Group IPT		Group	Face-to-face	Community	No	IPT Group: 1 h per week for 12 weeks, Peer Support Group: 20 min/week for 12 weeks
Forman et al. (2007) [54]	PP	6 months postpartum	IPT with mothers and their babies		Mother-infant	Face-to-face	Community	No	12 weeks of IPT
L. L. Gao et al., 2010 [55]	PN	over 28 weeks pregnant	Routine antenatal education & IPT-oriented childbirth education program	Small groups of no more than 10 people	Groups, Telephone	Combination Face-to-face (group) and one telephone follow-up call in the postpartum period (2 weeks)	Community	No	Intervention group received routine antenatal education [2 × 90-min sessions conducted by midwives, content: delivery process and childcare] & IPT-oriented childbirth psychoeducation program [Two 2-hr group sessions with one telephone follow-up in the postpartum period]
L. L. Gao et al. (2012) [56]	PN	over 28 weeks pregnant	Routine childbirth education program & IPT-oriented childbirth education program	Small groups of no more than 10 people	Groups, Telephone	Combination Face-to-face (group) and one telephone follow-up call in the postpartum period (2 weeks)	Community	No	Intervention group received routine antenatal education [2 × 90 min sessions conducted by midwives, content: delivery process and childcare] & IPT-oriented childbirth psychoeducation program [Two 90 min antenatal group sessions with one telephone follow up within 2 weeks after delivery]
L. L. Gao et al. (2012) [57]	PN	over 28 weeks pregnant	Routine antenatal childbirth education & IPT-oriented childbirth psychoeducation program	Small groups of no more than 10 people	Groups, Telephone	Combination Face-to-face, telephone	Community	No	Routine childbirth education classes (2–90-min sessions) & IPT-oriented childbirth psychoeducation program (Two 90 min antenatal group sessions with one telephone follow up within 2 weeks after delivery)
L. L. Gao et al. (2014) [58]	PN	over 28 weeks pregnant	Routine childbirth education program & IPT-oriented childbirth education program		Groups, Telephone	Combination Face-to-face (group) and one telephone follow-up call in the postpartum period (2 weeks)	Community	No	Intervention group received routine antenatal education [2 × 90 min sessions conducted by midwives, content: delivery process and childcare] & IPT-oriented childbirth psychoeducation program [Two 90 min antenatal group sessions with one telephone follow up within 2 weeks after delivery]
L. L. Gao et al. (2015) [59]	PP	2–3 days postpartum	Pamphlet on sources of assistance after discharge & IPT-oriented postnatal psychoeducation programme	Outcomes measured: Postpartum depressive symptoms, social support, and maternal role competence	Individual	Combination Face-to-face, telephone	Community	No	One 1-hr session (before hospital discharge) and a telephone follow-up within 2 weeks after discharge
Grote et al. (2004) [60]	PN	12–28 weeks pregnant	IPT-B (brief) & IPT-M (maintenance)	12 people who screened > 10 on the EPDS, IPT sessions scheduled as much as possible preceding or following their antenatal appt, depressed, low-income, minority women	Individual	Combination Face-to-face, telephone	Community	No	9 sessions (no timeframe for each session) (Pre-treatment engagement interview, 8 IPT-B [Brief] sessions, IPT-M [maintenance] sessions monthly up to 6 months [max: 6 sessions] Postpartum)
Grote et al. (2009) [61]	PN	10–32 weeks pregnant	IPT-B—multicomponent, enhanced, culturally relevant (reflected 7/8 components delineated in the culturally centered framework of Bernal and colleagues (1995))	EPDS ≥ 12, ≥18 years old, English speaking, low income. Cultural sensitivity and Culturally relevant additions integrated into IPT-B (free bus passes, childcare, facilitate access to social services—food, job training, housing, free baby supplies)	Individual	Combination—Face-to-face, telephone	Community	No	Pre-treatment engagement interview, 8—Brief IPT sessions (in-person, telephone), and bi-weekly or monthly IPT maintenance for up to 6 months post-baseline,
Grote et al. (2015) [62]	PN	12–32 weeks pregnant	MSS-Plus AND MOMCare—18 month collaborative care intervention stepped treatment approach (included initial pre-treatment engagement session, choice of IPT-B and/or pharmacotherapy, telephone plus in-person visits)	screened to include participants who had probable depression/dysthymia,	Individual	Combination Face-to-face, telephone (calls or texts)	Community (Public Health Centers, Patient’s home)	No	Pre-treatment engagement interview, 8—Brief IPT sessions every 1–2 weeks (in-person, telephone) across 3–6 months post-baseline, and monthly IPT maintenance for up to 18 months post-baseline, 60 min/session
Grote et al. (2017) [63]	PN	12–32 weeks pregnant	MOMCare—18-month collaborative care intervention, stepped treatment approach—women with less than 50% improvement in depressive symptoms by 6–8 weeks received a revised treatment plan	screened for depression, Patient Health Questionnaire-9 (PHQ-9) scoring ≥ 10, and screened for dysthymia: MINI	Individual	Combination—Face-to-face, telephone	Community (Public Health Centers, Patient’s home)	No	Pre-treatment engagement interview, 8—Brief IPT sessions every 1–2 weeks (in-person, telephone) across 3–4 months post-baseline, and monthly IPT maintenance for up to 18 months post-baseline, 60 min/session
Hajiheidari et al. (2013) [64]	PP	not specified	IPT—marriage	EPDS ≥ 14, and by the diagnosing review by a psychologist	Partners	Face-to-face	Community	Yes (scores not collected/analysed)	10—sessions/10 weeks
Kao et al. (2015) [65]	PN	20–35 weeks pregnant	IPT—Reach Out, Stand Strong, Essentials for new mothers (ROSE) & standard care	score of 27 or greater on a 17-item tool to assess PDD, low income	Group (3–5 people per group)	Face-to-face	Community (Groups at prenatal clinic, Booster at clinic or participant’s home)	No	4 sessions/60 min/4 weeks and one 50-min booster after delivery
Klier et al. (2001) [66]	PP	4–45 weeks postpartum	IPT		Combination (Individual and Group)	Face-to-face	Clinical	No	12 sessions: Individual (two 60-min pre-sessions), Group (nine 90-min weekly group sessions), Individual (one 60-min termination session)
Kozinszky, Dudas, Devosa, Csatordai, Tóth, et al. (2012) [67]	PN	25–29 weeks pregnant	Psychoeducation and psychotherapy for PPD utilizing IPT and CBT elements—each session ended with relaxation exercises		Group (max 15 per group)	Face-to-face	Community	Yes—allowed to attend	4 sessions—3-h—over 4 consecutive weeks
Lenze et al. (2015) [68]	PN	12–30 weeks pregnant	IPT-Dyad—two phases, antepartum phase based on brief, culturally relevant IPT developed by Grote 2008 (weekly sessions), postpartum phase (biweekly sessions then monthly)		Individual	Face-to-face	Community (Sessions offered at participant’s home, at the clinic, or at other convenient community location)	No	Antenatal—minimum dose 7 sessions—55% achieved minimum dose—sessions included an engagement session to explore views about depression, treatment, and barriers to care strategies of standard IPT. Postpartum—minimum dose of 8—71% achieved minimum dose—sessions were on maintaining interpersonal functioning, infant emotional development theory, and attachment theory
Lenze and Potts (2017) [69]	PN	12–30 weeks pregnant	Brief IPT engagement session and then 8 IPT sessions—those who completed all 9 sessions had access to maintenance sessions		Individual	Combination Face-to-face (participants had the option to receive brief-IPT over the phone)	Community (Sessions offered at participant’s home, at the research clinic, or at other convenient community location)	No	1 engagement session, 8 IPT sessions as described by Grote et al. 2004 (length of time for sessions not included)
Leung and Lam (2012) [70]	PN	14–32 weeks pregnant	IPT-oriented intervention		Group	Face-to-face	Community	No	4 weekly 1.5-h sessions/4 weeks
Moel et al. (2010) [71]	PP	Postpartum—not sure of timing	IPT	Sample from O’Hara study 2000	Individual	Face-to-face	Community (Therapist’s private practice clinics)	No	12 h over 12 weeks
Mulcahy et al. (2010) [72]	PP	less than 12 months postpartum	IPT	60% onset of current depression after the birth of the baby, 22% during pregnancy, 18% prior to conception	Combination (Individual, Group, partners)	Face-to-face	Clinical	Yes (evening session only)	11 sessions in total (2 individual, 8 group, 1 evening group for men only—each 2 h/session) over 8 weeks
Nylen et al. (2010) [73]	PP	6–24 months postpartum	IPT	Sample from O’Hara study 2000	Individual	Face-to-face	Community	No	12 h over 12 weeks (12—1-h sessions over 12 weeks)
O’Hara et al. (2000) [74]	PP	6–9 months postpartum	IPT	This sample also used in the Nylen study	Individual	Face-to-face	Clinical	No	12 h over 12 weeks
O’Hara et al. (2019) [75]	PP	within 6 months postpartum	IPT	Recruited from 2008 to 2013	Individual	Face-to-face	Clinical	No	12 individual 50-min sessions over 12 weeks
Pearlstein et al. (2006) [76]	PP	6 months postpartum	IPT	11 women picked IPT, 2 picked sertraline, and 10 picked sertraline and IPT	Individual	Face-to-face	Clinical (outpatient mental health setting)	No	IPT: 12–50-min sessions over 12 weeks,
Posmontier et al. (2016) [77]	PP	6 weeks–6 months postpartum	CNM-IPT (Certified Nurse-Midwives Telephone Administered Interpersonal Psychotherapy)		Individual	Telephone	Clinical	No	8 sessions lasting 50 min per session over a 12–week period
Posmontier et al. (2019) [78]	PP	1–6 months postpartum	IPT		Individual	Face-to-face	Clinical	No	Up to 8 × 50-min sessions
Reay et al. (2006) [79]	PP	less than 12 months postpartum	IPT-G (Group)		Group (with individual, partners)	Face-to-face	Community (local community centers)	Yes	2 individual sessions (pre-therapy, 6–week post-group appointment), 8 weekly group sessions at 2 h a session (delivered over 8 weeks), 2-h partners evening (midway through group sessions—weeks 3–7)
M. G. Spinelli (1997) [19]	PN	6–40 weeks pregnant	IPT for antenatal depression		Individual	Face-to-face	Clinical	No	16 weekly sessions, 50 min per session
Spinelli and Endicott (2003) [20]	PN	6–36 weeks pregnant	IPT for antenatal depression—bilingual (Spanish and English)	lower socioeconomic 50 started—25 in each group—ended with 17 in control group and 21 in treatment group	Individual	Combination Face-to-face, telephone (as needed)	Clinical and Community	No	16 weekly 45 min per session
Spinelli, Endicott, Leon, et al. (2013) [80]	PN	12–33 weeks pregnant	IPT for antenatal depression (bilingual) (breastfeeding education provided to 83% participants even though not mandatory)	Same sample as the Spinelli et al. 2013b	Individual	Combination Face-to-face, telephone (as needed)	Clinical and Community	No	12 weekly sessions—45 min per session
Spinelli, Endicott, and Goetz (2013) [81]	PN	12–33 weeks pregnant	IPT for antenatal depression—bilingual (Spanish and English)		Individual	Combination Face-to-face, telephone (as needed),	Clinical and Community	No	12 weekly sessions—5 min per session
Zlotnick et al. (2001) [82]	PN	12–32 weeks pregnant	IPT (Survival Skills for New Moms)	women receiving public assistance	Group	Face-to-face	Community	No	4–60-min sessions over 4 weeks
Zlotnick et al. (2006) [83]	PN	12–32 weeks pregnant	ROSE program IPT-based intervention & standard antenatal care	women receiving public assistance	Group (and Individual-booster)	Face-to-face	Community	No	four sessions 60 min group session over 4 weeks and a 50-min individual booster session after delivery
Zlotnick et al. (2011) [84]	PN	12–32 weeks pregnant	IPT—for Depression and PTSD	women with intimate partner violence—low-income	Individual	Face-to-face	Community	No	4–60-min sessions over 4 weeks, 1–60 min individual ‘booster’ session within 2 weeks of delivery
Zlotnick et al. (2016) [85]	PN	20–35 weeks pregnant	ROSE program IPT-based intervention—group & standard antenatal care	women receiving public assistance	Group (and Individual-booster)	Face-to-face	Community	No	4–90-min group sessions over a 4-week period, and a 50-min individual booster session 2 weeks after delivery

**Table 3:** Method of Assessment for Outcomes in Included Analyses.

Study	Type (Prevention or Treatment Study)	Assessment of Depressive Symptoms	Prevalence of Depressive Episodes	Assessment of Symptoms of Anxiety	Stress	Attachment	Quality of Life	Relationship Satisfaction/Quality	Adjustment	Social Support	Others
Bhat et al. (2017) [44]	Treatment	SCL-20	PHQ-9, MINI		PTSD-Checklist Civilian Version (PCL-C)				WSAS	WSAS	PES
Bowen et al. (2014)	Prevention	EPDS		STAI	CWS					MSSS	Satisfaction with Psychotherapy group: 1. What did you find most positive about the group? 2. What would you change in the group?
Brandon et al. (2012) [21]	Treatment	HAM-D EPDS, EPDS—Partner version	DSM-IV MDD, SCID-IV, HAM-D17					DAS	DAS		
Chen (2011) [46]	Treatment	EPDS	EPDS				GAF				
Chung (2015) [47]	Treatment	EPDS, HAM-D	EPDS = 22	HAM-A							
Clark et al. (2003) [48]	Treatment	CES-D, BDI	DSM-IV MDD		PSI			PCERA			BSID
Crockett et al. (2008) [49]	Prevention	EPDS	CSQ > 27, SCID-R		PSI				SAS-SR, PPAQ		
Deans et al. (2016) [50]	Treatment	BDI	SCID-II, EPDS	BAI	PSI	MAI					Infant Characteristics Questionnaire, Emotional Availability Scales (EAS)
Dennis et al. (2020) [51]	Treatment	EPDS > 12 eligible to be referred	SCID depression module. EPDS > 12.	STAI		ECR		DAS			Health service utilization and costs
Field et al. (2009) [52]	Treatment	CES-D	SCID-I	STAI	Cortisol samples (saliva)			The relationship questionnaire		SSQ-R	STAXI
Field et al. (2013) [53]	Treatment	CES-D	SCID-I	STAI	Cortisol samples (saliva)						STAXI
Forman et al. (2007) [54]	Treatment	IDD, HAM-D	IDD, SCID, HRSD		PSI	AQS					IBQ, CBQ, Maternal Responsiveness, Child Behaviour Problems—Child Behavior Checklist/2–3
L. L. Gao et al. (2010) [55]	Prevention	EPDS	EPDS ≥ 13					Satisfaction with Interpersonal Relationships Scale			GHQ
L. L. Gao et al. (2012) [56]	Prevention	EPDS, GHQ	EPDS ≥ 13							PSSS	PSOC—with Efficacy (PSOC-E). GHQ
L. L. Gao et al. (2012) [57]	Prevention									PSSS	Qualitative interviews—looking at close ended questions of the Program Satisfaction Questionnaires
L. L. Gao et al. (2014) [58]	Prevention									PSSS	PSOC—with Efficacy (PSOC-E)
L. L. Gao et al. (2015) [59]	Prevention	EPDS	EPDS ≥ 13							PSSS	PSOC—with Efficacy (PSOC-E)
Grote et al. (2004) [60]	Treatment	EPDS, BDI, HAM-D	EPDS > 10, DIS	BAI				IIP	SAS, PPAQ	Medical Outcomes Study Social Support Survey	satisfaction with each social support, participants completed a 4-item treatment satisfaction survey and 5-point Likert scale on how positive they felt about their pregnancy (after each session)
Grote et al. (2009) [61]	Treatment	EPDS, BDI, SCID	EPDS ≥ 12	BAI					SAS, PPAQ		CAGE-AID, MINI
Grote et al. (2015) [62]	Treatment	Hopkins Symptom Checklist SCL-20	PHQ-9 ≥ 10 and at least five symptoms scored as ≥2 with one cardinal symptom on the PHQ-9, plus a functional impairment to include participants with probable MDD, MINI-International Neuropsychiatric Interview (MINI) to include participants with probable dysthymia	PHQ	PCL-C			RQ	WSAS		CAGE-AID, MINI, childhood trauma—Childhood Trauma Questionnaire
Grote et al. (2017) [63]	Treatment	SCL-20	PHQ-9, MINI	PHQ	PCL-C						CAGE-AID, MINI, SCL-20 (Depression-free Days (DFDs)), Costs for MOMCare intervention, CSI
Hajiheidari et al. (2013) [64]	Treatment	EPDS, BDI-II	EPDS ≥ 14 (used for primary screening only)					Revised Double Adaptive Score (Marriage Adaptive)			EPDS ≥ 14 and by the diagnosing review by a psychologist
Kao et al. (2015) [65]	Treatment	Predictive Index of PPD, EPDS	Predictive Index of PPD—score of 27 or higher (high-risk status)						SAS		Breast feeding—initiation and duration
Klier et al. (2001) [66]	Treatment	HAM-D-21, EPDS	SCID-I, HAM-D-21 > 13.					DAS	DAS		Inventory of Interpersonal Problems (IIP) (German version), SCID-II used to diagnose Axis II disorders
Kozinszky, Dudas, Devosa, Csatordai, Tóth, et al. (2012) [67]	Prevention	LQ ≥ 12									Additional structured questions exploring sociodemographic, economic, and psychological risk factors
Lenze et al. (2015) [68]	Treatment	EPDS	EPDS > 12, SCID—Axis I		PSI					SSQR	Infant-Toddler Social and Emotional Assessment, Client Satisfaction Questionnaire (acceptability)
Lenze and Potts (2017) [69]	Treatment	EPDS	EPDS ≥ 10, SCID	Brief-STAI				ECR-R		SSQR	DLC, CSQ
Leung and Lam (2012) [70]	Prevention	EPDS	EPDS < 12		PSS			Relationship Efficacy Measure			perceived ability to cooperate in childcare, 4-item subjective happiness scale
Moel et al. (2010) [71]	Treatment	SCID, BDI, HAM-D	IDD, SCID-I					DAS	DAS		LIFE-II
Mulcahy et al. (2010) [72]	Treatment	HAM-D, EPDS, BDI	MCMI-III, HAM-D ≥ 14			MAI		DAS		ISEL	
Nylen et al. (2010) [73]	Treatment	BDI, HAM-D	IDD, SCID, HAM-D scores ≥ 12								LIFE-II
O’Hara et al. (2000) [74]	Treatment	SCID, HAM-D) (≥12), BDI	IDD, SCID					DAS	SAS-SR, PPAQ, DAS		HAM-D adding items on hypersomnia, hyperphagia and weight gain
O’Hara et al. (2019) [75]	Treatment	BDI, EPDS, PHQ-9 replaced the EPDS	SCID, HAM-D ≥ 15	Inventory of Depression and Anxiety Symptoms, General depression scale					PPAQ		Clinical Global Impressions-Severity of Illness and Improvement scales
Pearlstein et al. (2006) [76]	Treatment	BDI, HAM-D, EPDS	SCID, BDI ≥25, HAM-D ≥ 14, EPDS								
Posmontier et al. (2016) [77]	Treatment	HAM-D, EPDS	EPDS > 9, MINI—met criteria for MDD			Mother-to-Infant Bonding Scale			DAS	SSQ	GAF, CSQ-8, MINI, IAQS
Posmontier et al. (2019) [78]	Treatment	EPDS	EPDS score of 10–18 for inclusion						PPAQ		CSQ-8
Reay et al. (2006) [79]	Treatment	HAM-D, EPDS, BDI	EPDS >13						SAS		Patient Satisfaction Survey (developed for this study)
M. G. Spinelli (1997) [19]	Treatment	HAM-D, EPDS, BDI	SCID, HAM-D ≥ 12								Serum thyroid function tests, Clinical Global Impression (global ratings of symptom severity and improvement)
Spinelli and Endicott (2003) [20]	Treatment	HAM-D, BDI, EPDS	SCID, HAM-D ≥ 12			Maudsley Mother Infant Interaction Scale					Assessment of Mood Change (weekly), Clinical Global Impression (global ratings of symptom severity and improvement)
Spinelli, Endicott, Leon, et al. (2013) [80]	Treatment	HAM-D, EPDS	SCID, HAM-D ≥ 12			Postpartum Bonding Questionnaire					Breastfeeding, SCID for DSM-IV to rule out comorbid diagnosis, Clinical Global Impression (global ratings of symptom severity and improvement)
Spinelli, Endicott, and Goetz (2013) [81]	Treatment	HAM-D, EPDS	SCID, HAM-D ≥ 12			Maternal Fetal Attachment Scale					SCID for DSM-IV to rule out comorbid diagnosis, Clinical Global Impression (global ratings of symptom severity and improvement)
Zlotnick et al. (2001) [82]	Prevention	BDI	SCID								
Zlotnick et al. (2006) [83]	Prevention	BDI, LIFE	CSQ > 27						Range of Impaired Functioning Tool		SCID for DSM-IV-NP Axis 1 to rule out comorbid diagnosis,
Zlotnick et al. (2011) [84]	Prevention	EPDS, PSR, LIFE									Revised Conflict Tactic Scale (CTS2)—assessed for IPV in last year for inclusion The Davidson Trauma Scale Criterion A from the PTSD module of the SCID-NP for DSM-IV—assessed for history of trauma, SCID-NP for DSM-IV Axis I—assessed for affective d/o, PTSD, SUD for exclusion
Zlotnick et al. (2016) [85]	Prevention	LIFE, PSR	CSQ > 27								SCID for DSM-IV-NP to exclude those with comorbid diagnosis, Treatment Services Review (TSR)

**Table 4:** Effective Public Health Practice Project (EPHPP) Quality Assessment Tool.

Study	Selection Bias	Study Design	Confounders	Blinding	Data Collection Methods	Withdrawal or Drop-Outs	Intervention Integrity	Analysis	Overall Rating
Bhat et al. (2017) [44]	1	1	3	3	1	2	2	2	2
Bowen et al. (2014) [45]	3	3	3	3	1	1	1	2	3
Brandon et al. (2012) [21]	3	3	3	3	1	1	1	2	3
Chen (2011) [46]	2	3	3	3	1	3	3	2	3
Chung (2015) [47]	3	3	3	3	2	1	1	2	3
Clark et al. (2003) [48]	1	2	1	2	1	2	1	2	2
Crockett et al. (2008) [49]	1	1	2	2	1	1	1	2	1
Deans et al. (2016) [50]	3	3	3	3	1	1	1	2	3
Dennis et al. (2020) [51]	1	1	1	2	1	2	1	1	1
Field et al. (2009) [52]	2	1	1	2	1	2	1	2	2
Field et al. (2013) [53]	2	1	1	2	1	1	1	2	1
Forman et al. (2007) [54]	2	1	1	1	1	2	1	2	1
L. L. Gao et al. (2010) [55]	1	1	1	2	1	1	1	2	1
L. L. Gao et al. (2012) [56]	1	1	1	2	1	1	1	1	1
L. L. Gao et al. (2012) [57]	3	3	3	3	2	1	1	1	3
L. L. Gao et al. (2014) [58]	3	3	2	3	2	1	1	1	3
L. L. Gao et al. (2015) [59]	1	1	1	2	1	1	1	1	1
Grote et al. (2004) [60]	1	1	1	1	1	1	1	1	1
Grote et al. (2009) [61]	1	1	1	3	1	1	1	1	1
Grote et al. (2015) [62]	1	1	1	1	1	1	1	1	1
Grote et al. (2017) [63]	1	1	1	1	1	1	1	1	1
Hajiheidari et al. (2013) [64]	3	1	3	3	1	3	1	2	3
Kao et al. (2015) [65]	1	1	1	2	1	2	1	1	1
Klier et al. (2001) [66]	2	2	3	3	3	3	2	3	3
Kozinszky, Dudas, Devosa, Csatordai, Tóth, et al. (2012) [67]	2	1	1	1	1	1	2	1	1
Lenze et al. (2015) [68]	2	2	2	3	1	1	1	1	2
Lenze and Potts (2017) [69]	2	2	2	3	1	1	1	1	2
Leung and Lam (2012) [70]	2	2	2	2	1	1	1	1	2
Moel et al. (2010) [71]	1	1	2	2	1	1	1	2	2
Mulcahy et al. (2010) [72]	1	1	1	2	1	1	1	1	1
Nylen et al. (2010) [73]	1	1	1	2	1	1	1	1	1
O’Hara et al. (2000) [74]	1	1	1	2	1	1	1	1	1
O’Hara et al. (2019) [75]	1	1	1	2	1	1	1	1	1
Pearlstein et al. (2006) [76]	1	3	2	3	2	1	2	2	2
Posmontier et al. (2016) [77]	2	2	2	2	2	2	1	2	2
Posmontier et al. (2019) [78]	1	3	2	3	1	2	2	2	3
Reay et al. (2006) [79]	2	2	2	3	1	1	1	2	2
M. G. Spinelli (1997) [19]	3	3	3	3	1	2	3	2	3
Spinelli and Endicott (2003) [20]	2	1	2	3	1	1	2	2	2
Spinelli, Endicott, Leon, et al. (2013) [80]	1	1	1	3	1	2	2	2	2
Spinelli, Endicott, and Goetz (2013) [81]	1	1	1	3	1	2	2	2	2
Zlotnick et al. (2001) [82]	3	2	1	3	1	1	1	2	3
Zlotnick et al. (2006) [83]	3	1	1	3	1	1	1	1	3
Zlotnick et al. (2011) [84]	1	2	2	3	1	1	1	2	2
Zlotnick et al. (2016) [85]	1	1	1	1	1	1	1	1	1

1 = Strong, 2 = Moderate, and 3 = Weak.

## Supplementary Materials

The following are available online at https://www.mdpi.com/1660-4601/17/22/8421/s1.

## List of Abbreviations

ANRQ	Antenatal Risk Questionnaire
AQS	Attachment Style Questionnaire
BAI	Beck Anxiety Inventory
BDI	Beck Depression Inventory
BDI-II	Beck Depression Inventory
Brief-STAI	Brief State-Trait Anxiety Inventory
CAGE-AID	Questionnaire for Drug and Alcohol Addiction Screening
CBT	Cognitive Behavioral Therapy
CBQ	Child Behavior Questionnaire
CES-D	Centre for Epidemiological Studies Depression Scale
CIDI	Composite International Diagnostic Interview
CNM-IPT	Certified Nurse-Midwife Telephone Administered Interpersonal Psychotherapy
CSQ	Cooper Survey Questionnaire
CTS2	Revised Conflict Tactic Scale
CWS	Cambridge Worry Scale
DAS	Dyadic Adjustment Scale
DASS-21	Depression, Anxiety, and Stress Scale—21 items
DIS	Diagnostic Interview Schedule
DLC	Difficult Life Circumstances
DSM-IV	Diagnostic and Statistical Manual of Mental Disorders, fourth edition
DSM-IV-NP	Diagnostic and Statistical Manual of Mental Disorders, fourth edition, non patient version
EAS	Emotional Availability Scale
ECR	Experiences in Close Relationships Scale
ECR-R	Experiences in Close Relationships Revised Scale
EPDS	Edinburgh Postnatal Depression Scale
EPDS-P	Edinburgh Postnatal Depression Scale for Partners
EFCT	Emotional Focused Couples Therapy
EPHPP	Effective Public Health Practice Project
GAF	Global Assessment of Functioning
GHQ	General Health Questionnaire
HADS	Hospital Anxiety and Depression Scale
HAM-A	Hamilton Anxiety Rating Scale
HAM-D	Hamilton Depression Rating Scale
HSC	Hopkins Symptoms Checklist
HRSD	Hamilton Rating Scale for Depression (also written as HAM-D)
IBQ	Infant Behavior Questionnaire
ICD	International Classification of Disease
ICMJE	International Committee of Medical Journal Editors
IDD	Inventory of Diagnose Depression
IIP	Inventory of Interpersonal Problems
IPT	Interpersonal Psychotherapy
IPT-G	Interpersonal Psychotherapy for Groups
LIFE	Longitudinal Interval Follow-up Evaluation
LIFE-R	Longitudinal Interval Follow-up Evaluation Revised
LQ	Leverton Questionnaire
MAI	Maternal Attachment Inventory
MCMI-II	Millon Clinical Multiaxial Inventory
MDD	Major Depressive Disorder
MFB	Mindfulness Based
MINI	MINI-International Neuropsychiatric Interview
M-ITG	Mother-Infant Therapy Group
MSSS	Maternity Social Support Scale
OT	Open Trial
PLC-C	Post-traumatic Stress Disorder Checklist–Civilian Version
PES	Peripartum Events Scale
PHQ	Patient Health Questionnaire
PHQ-9 Questionnaire Revised	Patient Health
PPAQ	Postpartum Adjustment Questionnaire
PPD	Postpartum Depression
PRISMA-P	Preferred Reporting Items for Systematic Reviews and Meta-analyses Protocols
PSI	Parenting Stress Index
PSOC	Parenting Sense of Competence Scale
PSOC-E	Parenting Sense of Competence with Efficacy Scale
PSR	Psychiatric Status Rating
QRT	Quasi-Randomized Controlled Trial
RCT	Randomized Controlled Trial
ROSE	Reach out, Stand Strong
SAS	Social Adjustment Scale
SAS-SR	Social Adjustment Scale Self Report
SCID	Structured Clinical Interview for DSM
SCID-I	Structured Clinical Interview for DSM I
SCID-IV	Structured Clinical Interview for DSM IV
SCL-90	Symptoms Checklist 90 Questions
SSQ-R	School Situations Questionnaire-Revised
STAI	State-Trait Anxiety Inventory
STAXI	State-Trait Anger Expression Inventory
SUD	Substance Use Disorders
TAU	Treatment as Usual
TSR	Treatment Services Review
WSAS	Weinberg Screening Affective Scales
WLC	Waitlist Control
