# Supplementary material for: Interpersonal Psychotherapy to Reduce Psychological Distress in Perinatal Women: A Systematic Review
Source: Int J Environ Res Public Health. 2020 Nov 13;17(22):8421. doi: 10.3390/ijerph17228421 (PMC7697337; doi:10.3390/ijerph17228421)
Supplement: Supplementary file 1 [file ijerph-17-08421-s001.zip › ijerph-961179-supplementary.pdf]

Appendix A Medline Search Strategy

Interpersonal Psychotherapy to Reduce Psychological Distress in Perinatal Women: A Systematic Review

Medline search strategy was adapted for the other databases. Keywords were constant across databases and subject headings were relevant to each specific database

Database(s): **Ovid MEDLINE(R) and Epub Ahead of Print, In-Process & Other Non-Indexed Citations and Daily**

Search Strategy:

#	**Searches**
1	exp Prenatal Care/
2	exp Postnatal Care/
3	exp Postpartum Period/
4	exp Peripartum Period/
5	exp Pregnancy/
6	(perinatal or prenatal or antenatal or peri-natal or pre-natal or ante-natal or postnatal or post-natal).tw,kf.
7	(peripartum or peri-partum or intrapartum or intra-partum or antepartum or ante-partum or postpartum or post-partum or puerperium or puerperal).tw,kf.
8	pregnanc*.tw,kf.
9	pregnant.tw,kf.
10	or/1-9
11	((interpersonal or inter-personal) adj2 (therap* or psychotherap* or psycotherap* or psycho-therap* or counsel*)).tw,kf.
12	ipt.tw,kf.
13	ipt-g.tw,kf.
14	or/11-13
15	10 and 14
